# Cerebral amyloid angiopathy: subtypes, treatment and role in cognitive impairment

**DOI:** 10.1007/s00415-017-8608-7

**Published:** 2017-09-06

**Authors:** D. McLauchlan, G. A. Malik, N. P. Robertson

**Affiliations:** Division of Psychological Medicine and Clinical Neuroscience, Department of Neurology, Cardiff University, University Hospital of Wales, Heath Park, Cardiff, CF14 4XN UK

## Introduction

Cerebral amyloid angiopathy (CAA) is characterised by the build-up of β-amyloid in the lining of leptomeningeal and cortical arterioles and is responsible for cerebrovascular disease in the form of lobar intracerebral haemorrhage, microbleeds and superficial siderosis. As a result CAA has been thought of as an independent contributor affecting normal brain function and especially cognition in the elderly. Prominent cerebral amyloid angiopathy is often observed in the brains of elderly individuals and is almost universally found in patients with Alzheimer’s disease. Despite the prevalence of the condition and associated morbidity, no effective treatments exist for the non-inflammatory subtype. However, accurate diagnosis of clinical subtypes and knowledge of CAA’s contribution to Alzheimer’s pathology may allow the identification of novel treatment targets for CAA and Alzheimer’s.

Current research suggests a number of promising therapeutic avenues including reducing amyloid production, increasing rate of clearance of amyloid from blood vessels and reducing the toxic effects of amyloid on the blood vessels. However, despite the apparent appropriateness of these approaches and a clear clinical need in an ageing population, clinical trials in CAA remain sparse.

This month’s journal club examines three articles on CAA exploring its role in cortical atrophy, potential treatment avenues and classification of CAA using a clinico-radiological diagnostic framework.

## Passive immunotherapy targeting amyloid-beta reduces cerebral amyloid angiopathy and improves vascular reactivity

Accumulation of β amyloid in leptomeningeal vessels and cortical arterioles is the hallmark of cerebral amyloid angiopathy, this causes vessel stiffening, and vessel wall rupture. Alzheimer’s disease amyloid plaques are composed of β-amyloid 42, whilst CAA results from accumulation of the β-amyloid 40, although β-amyloid 40 may migrate to the vasculature from parenchymal plaques. Recent work has shown that inefficient clearance of amyloid, rather than excess production is the primary contributor to β amyloid accumulation. Early work using a variety of approaches to increase clearance of β-amyloid, suggested a possible excess of micro-haemorrhages in animal models. A recent study from this group showed that chronic administration of high dose Ponezumab (a monoclonal antibody targeting β-amyloid) in an older group of transgenic mice did not increase micro-haemorrhage. This study tested the efficiency of β-amyloid clearance from the vasculature, and the consequences of β-amyloid clearance on vascular reactivity.

Transgenic mice between 6 and 22 months were used. These were from a variety of strains—APP knock-in, compound knock-in APP-PS1 and APP PSdE9 compound knock-in animals. Multi-photon imaging used methoxy x-034 to label amyloid and pre-imaging rhodamine injection to track blood vessels. Animals were imaged on a monthly basis. The brains were then removed and lysed, using centrifugation, the vascular tissue was separated from cortical regions and myelin. ELISA was used to measure β-amyloid quantitatively, whilst immunohistochemistry was used to study the distribution and number of vessels affected. Microdialysis was employed to measure the burden of β-amyloid in the interstitial fluid. Cerebral vessels were exposed, and arteriolar responses to acetylcholine and hypercapnia was recorded by observers using a light microscope.

Immunohistochemistry showed a reduction in cerebrovascular β-amyloid burden, as did the ELISA and multi-photon imaging. Parenchymal amyloid burden was unchanged. The microdialysis showed an increase in interstitial fluid β-amyloid, but not serum β-amyloid, following Ponezumab administration, suggesting a mobilisation of β-amyloid from the vasculature into the interstitial fluid as the mechanism of β-amyloid clearance. Finally, administration of Ponezumab improved vascular reactivity to acetylcholine and hypercapnia.

### Comments

This study extends the previous work, demonstrating an improvement in vascular reactivity and a reduction in cerebral amyloid burden following Ponezumab administration. Furthermore, the work strongly suggests the mechanism of amyloid reduction, is by mobilising the interstitial fluid pool of β amyloid. However, the study was mechanistic: the treatment was not compared with a control group, furthermore there were no assessments of cerebrovascular injury or cognitive change, leaving unanswered questions over the clinical relevance of vascular β-amyloid clearance.

*Bales KR et al. Brain (2016) 139; 563*–*577*

## Cortical atrophy in hereditary and sporadic forms of cerebral amyloid angiopathy: an observational case–control study

Cortical injury mediates the cognitive impairment of Alzheimer’s disease and other neurodegenerative disorders. Apparent cortical atrophy is often found in the brains of patients suffering from subcortical vascular injury. What is not clear is whether this atrophy is artefact from sulcal widening secondary to subcortical atrophy, or if there is both cortical and sub-cortical damage from small vessel disease. CAA exerts its most significant effects at the subcortical level, causing micro-vascular damage and consequent ischaemia. Sporadic CAA is associated with subcortical vascular damage, but also parenchymal amyloid deposition: this dual pathology precludes assessment of subcortical vascular disease in isolation. This study recruited a group with a genetic form of CAA [hereditary cerebral haemorrhage with amyloidosis—Dutch type (HCHWA-D)], to study the effects of subcortical vascular disease on cortical thickness. Cortical thickness was also assessed in a group with sporadic CAA, Alzheimer’s disease and a control group. Further imaging measures were taken to delineate the relationship between micro-vascular disease, vascular reactivity and cortical thickness.

Genetically confirmed subjects carrying the mutation in codon 693 of the amyloid precursor protein gene were recruited from Leiden University and Massachusetts General Hospital. 26 subjects with HCHWA-D were recruited and compared with 28 age-matched controls in Leiden, in addition to 63 non-demented patients with CAA, 189 healthy controls and 63 patients with Alzheimer’s disease from the US centre. Freesurfer cortical parcellation software was used to measure cortical thickness on images derived from FLAIR and T1 MPRAGE sequences. BOLD sequences during a standardised visual stimulus were used to assess vascular reactivity. All images were derived on 3T machines.

HCHWA-D subjects had thinner cortices than healthy controls, but hippocampal volume did not differ between the groups. Patients with sporadic CAA also had more marked cortical thinning when compared with a control group, particularly in the occipital, temporal, posterior parietal and medial frontal regions. These results were robust even when CAA patients with lobar haemorrhage were excluded. The Alzheimer’s disease group had more marked cortical thinning and hippocampal volume loss than the healthy controls and the CAA group. The BOLD measures correlated with cortical thickness in the HCHWA-D group, and remained significant even after adjusting for demographic variables, white matter disease, haemorrhage and microbleeds. Furthermore, the vascular reactivity measure mediated the case status relationship with cortical thinning: when vascular reactivity was added to the statistical model, case status became non-significant, demonstrating that vascular reactivity mediates the cortical thinning seen in the HCHWA-D subjects.

### Comments

The unique disease model of a purely vasculopathic form of CAA used in this elegant study has provided evidence of cortical thinning independent of Alzheimer pathology, and supports the hypothesis that vascular amyloid is an independent contributor to cortical atrophy. Furthermore, these findings are generalisable to sporadic CAA. This study lends support to the suggestion that CAA and small vessel disease is an important contributory factor in age-associated cognitive impairment and suggests a potentially modifiable pathway for future therapeutic research.

*Fotiadis P et al. Lancet Neurol. (2016); 15(8): 811*–*81*

## Validation of clinico-radiological criteria for the diagnosis of cerebral amyloid angiopathy: related inflammation

Cerebral amyloid angiitis (CAAri) is an inflammatory variant of CAA that responds to immunosuppressive therapy. Unfortunately proving the diagnosis often necessitates brain biopsy which carries a risk of significant morbidity and mortality. This study assessed the sensitivity and specificity of pre-specified clinico-radiological criteria for diagnosis of CAAri in a group of biopsy proven CAAri and CAA, to see if brain biopsy may be avoided in selected patients.

The criteria were: (1) clinical presentation with headache, altered consciousness, neuropsychiatric abnormalities, focal deficits and seizures, (2) patchy, confluent, subcortical T2/FLAIR lesions without meningeal involvement, (3) multiple micro-haemorrhages or superficial siderosis, and prior lobar haemorrhage, (4) absence of any other cause. ‘Probable CAAri’ cases had asymmetrical white matter change, whilst ‘Possible CAAri’ cases only required subcortical white matter change. The medical records of patients admitted to Massachusetts General Hospital between 1995 and 2013 were reviewed for a diagnosis of biopsy proven CAAri. 22 cases were found, 7 were excluded, as they did not have the relevant MR sequences. 2 further cases were identified from a biomarker study. The cases were compared with 37 controls who had a brain biopsy and who satisfied the Boston criteria for CAA. The control group was subdivided into those with (21) and without (16) intracerebral haemorrhage. Imaging was reviewed by a neurologist blind to case–control status, scans were reviewed by a second neurologist to verify accuracy.

The inter-rater reliability of the imaging rating was high (*Ҡ* 0.8). In the CAAri group 82% met the criteria for ‘Probable CAAri’ and ‘Possible CAAri’, in the control group with lobar haemorrhage, 5% met the ‘Possible CAAri’ and none met the ‘Probable CAAri’ criteria. The control group without haemorrhage, had a higher percentage of cases meeting criteria for Possible CAAri (69%) and 1 case met the ‘Probable CAAri’ criteria. The criteria had an overall sensitivity of 82% and specificity of 68%. Diffusion weighted imaging and contrast-enhanced imaging did not significantly differ between the groups. Intriguingly the control participant rated as ‘Probable CAAri’ was given high dose steroids and improved markedly, raising the possibility his brain biopsy may have missed the inflammatory region.

### Comments

The authors identify limitations in the retrospective design of the study and small case numbers. As a result, the classification system proposed may not be yet ready for general use, but the data presented suggests that in CAA patients with lobar haemorrhage a reliable diagnosis of CAA-ri might be reached from basic clinical and magnetic resonance imaging information alone. However, in those without haemorrhage, these criteria may not be so reliable. Furthermore a case could be made for the empirical use of immunosuppression without brain biopsy in selected patients.

*Auriel E et al. JAMA Neurol. (2016);73(2):197*–*202*

